# Immunomodulatory Effects of Recombinant *Mycobacterium smegmatis* Expressing Antigen-85B Epitopes in Infected J774A.1 Murine Macrophages

**DOI:** 10.3390/pathogens9121000

**Published:** 2020-11-29

**Authors:** Nur-Ayuni Kadir, Armando Acosta, Maria E. Sarmiento, Mohd-Nor Norazmi

**Affiliations:** 1 School of Biomedicine, Faculty of Health Sciences, Universiti Sultan Zainal Abidin, Kuala Nerus 21300, Terengganu, Malaysia; 2 School of Health Sciences, Universiti Sains Malaysia, Kubang Kerian 16150, Kelantan, Malaysia; mari@usm.my (M.E.S.); norazmimn@usm.my (M.-N.N.)

**Keywords:** tuberculosis, BCG, mycobacteria, *M. tuberculosis*, *M. smegmatis*, antigen-85B

## Abstract

Tuberculosis (TB) causes more than 1.5 million deaths each year, remaining a significant global health problem. *Mycobacterium smegmatis* (*M. smegmatis*) and *Mycobacterium tuberculosis* (*M. tuberculosis*) share features, which support the use of the former use in new generation TB vaccine development. In a previous study, the specific humoral and cellular immunogenicity of a recombinant *M. smegmatis* strain expressing epitopes from *M. tuberculosis* Ag85B protein (rMs064), was demonstrated in mice. In the current study, the immunomodulatory capacity of rMs064 was determined in a J774A.1 murine macrophage cell line. To determine the immunomodulatory effect of rMs064 in J774A.1 macrophages, the expression of inducible nitric oxide synthase (iNOS) and production of nitric oxide (NO) was evaluated. The expression of activation surface markers (MHC-II, CD40, CD80 and CD86) and the production of cytokines (IL-1β, TNF-α, IL-12p70 and IL-6) was also determined in rMs064 infected J774A.1 macrophages. Our findings showed the ability of rMs064 to induce substantial increases in macrophage activation markers expression; MHC class II and CD40, compared with *M. smegmatis t*ransformed with the empty vector (rMs012) and uninfected cells. rMs064 induced significant increases in IL-12p70 compared to uninfected cells. The expression of iNOS and CD86, and the production of IL-1β, and TNF-α were increased in rMs064 and rMs012, compared to uninfected cells. rMs064 demonstrated its immunomodulatory ability by stimulating the innate immune response, which supports its further evaluation as a TB vaccine candidate.

## 1. Introduction

Tuberculosis (TB) remains a significant global health issue that causes more than 1.5 million deaths each year [1].

*Mycobacterium smegmatis* (*M. smegmatis*) is a rapidly growing, non-pathogenic specie, which shares many genetic, functional and antigenic characteristics with *Mycobacterium tuberculosis* (*M. tuberculosis*), supporting its use in new generation TB vaccine development [2,3,4,5,6,7,8].

The innate immune response plays a crucial role in the host defense against mycobacteria. Innate immune cells, such as macrophages, elicit immune responses and act as a central player to further activate the more vigorous and specific adaptive immune response [9]. The macrophage immune activation involves phagocytosis and activation of reactive oxygen and nitrogen intermediates as a pathogen clearance mechanism. Cells from the innate immune system, including macrophages, recognize through pattern recognition receptors (PRRs) the pathogen-associated molecular patterns (PAMPs). PAMPs are microbial molecular signatures, such as Toll-like receptors (TLRs), which upon interaction with PRRs, activate the transcription of NF-κB. The increase in NF-κB transcription induces the production of cytokines, key elements in the innate immune response [10]. The recognition of mycobacterial TLRs, mainly associated with the mycobacteria cell wall, activate intracellular signaling cascades, which eventually leads the activation of NF-κB transcription with the production of pro- and anti-inflammatory cytokines and chemokines. The type of signaling cascade induced depends mainly on the type of PRRs that recognizes components of mycobacteria [11,12,13].

Ag85 complex (85A, 85B, and 85C) consists of highly homologous 30–32 kDa culture filtrate proteins (CFPs) of *M. tuberculosis* [14]. These antigens are associated with mycolyl-transferase activity in vitro and catalyze the synthesis of the glycolipids of the mycobacterial cell wall [15]. Ag85 belongs to the fibronectin-binding proteins, one of the critical mycobacterial virulence factors, and contribute to the bacterial attachment, invasion, and dissemination [16]. Fibronectin plays an important role in bacteria–host interactions during infection by binding to components of the microbial surface such as Ag85 [17]. Ag85B is one of the dominant proteins secreted by all mycobacterial species and has been shown to induce Th1 cytokines in mice and humans [18]. In experimental animal models, Ag85B has been shown to induce robust cellular and humoral immune responses and protection [19,20,21,22].

In a previous study, a recombinant *M. smegmatis* expressing epitopes from Ag85B (rMs064) was obtained and its specific cellular and humoral immunogenicity was demonstrated in mice [21].

In the current study the immunomodulatory capacity of rMs064 was demonstrated after infection of a J774A.1 murine macrophage cell line.

## 2. Results

### 2.1. Phagocytic Uptake of rMs064 and rMs012 by J7474A.1 Macrophages

**Figure 1:**
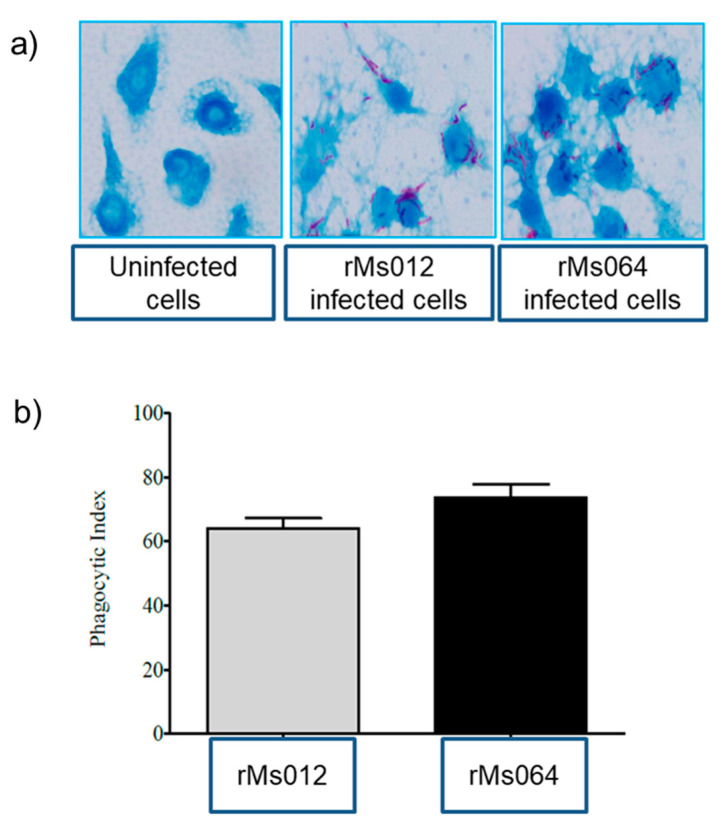
Phagocytic activity of J774A.1 macrophages infected with rMs064 and rMs012. (**a**) Comparison of morphology of rMs064 or rMs012 infected macrophages. Ziehl–Neelsen staining (100× magnification). (**b**) Phagocytic activity of rMs064 and rMs012 infected macrophages (4 h post-infection). Multiplicity of infection (10:1). Data represent the mean phagocytic index ± SEM from three independent experiments. Statistical analysis was performed by Student’s t-test.

Microscopic examination of macrophages after infection showed that both rMs064 and Ms012 were efficiently phagocytosed by the macrophages. No apparent change in the morphology of the infected macrophages and mycobacteria was observed. Figure 1a shows a representative image of infected and uninfected macrophages. Macrophages infected with rMs064 or rMs012 showed no significant difference in phagocytic index (Figure 1b).

### 2.2. Production of Nitric Oxide and Inducible Forms of Nitric Oxide Synthase (iNOS) by Infected Macrophages

**Figure 2:**
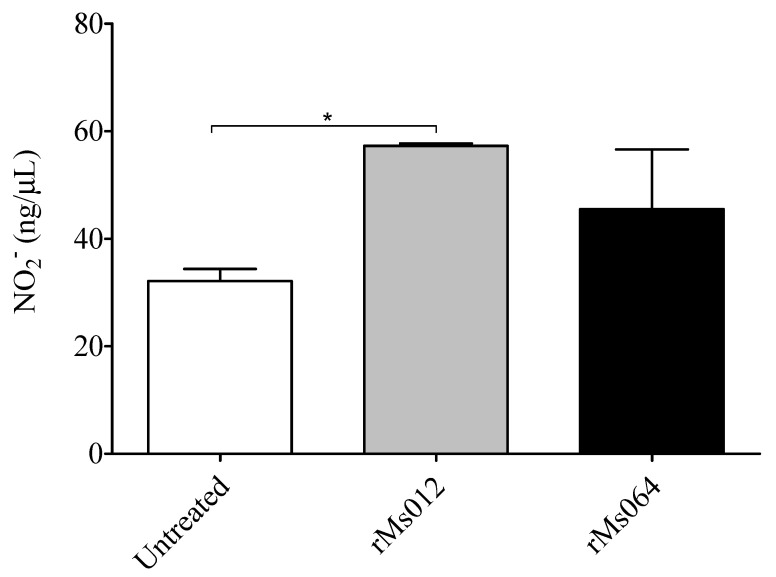
Production of nitric oxide by rMS064 or rMs012 infected macrophages (24 h post-infection). Data represent the mean concentration ±SEM of nitric oxide from three independent experiments. Statistical analysis was performed by one-way analysis of variance (ANOVA) followed by the Bonferroni multiple comparison test. * *p* < 0.05.

NO_2_ production was detected in all culture supernatants of infected and uninfected macrophages. rMs064 infected macrophages produced no significant increase in the level of NO_2_ at 24 h post-infection compared to rMs012 or uninfected cells. In contrast, rMs012 infected macrophages showed significant increase in the NO_2_ production compared to uninfected cells (Figure 2).

**Figure 3:**
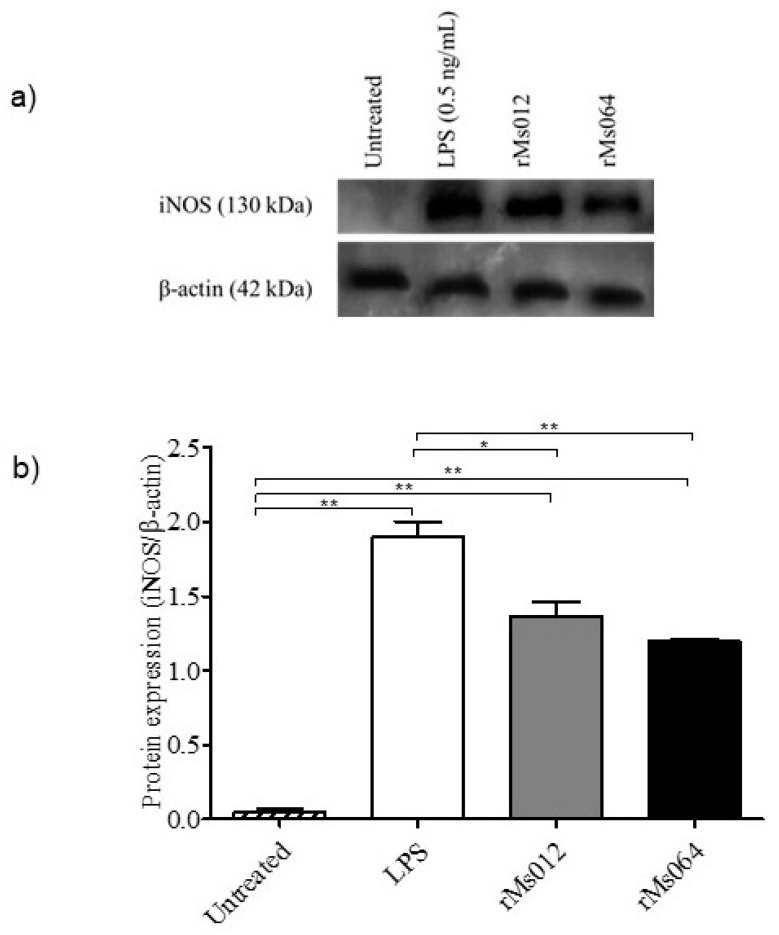
Expression of nitric oxide synthase (iNOS) by rMS064 and rMs012 infected macrophages (24 h of-incubation). (**a**) Western blot of representative result for iNOS and β-actin. Lipopolysaccharide (LPS) treatment (0.5 μg/mL) served as positive control for iNOS production. (**b**) Quantification of iNOS protein intensity relative to β-actin. Data represent the mean relative intensity of iNOS ±SEM from three independent experiments. Statistical analysis was performed by one-way analysis of variance (ANOVA) followed by the Bonferroni multiple comparison test. * *p* < 0.05 and ** *p* < 0.001.

The level of iNOS production was analyzed in macrophages infected with rMs064 or rMs012 at 24 h post-infection. Figure 3a,b shows the expression of iNOS in rMs064 or rMs012 infected macrophages. iNOS expression in rMs064- and rMs012-infected macrophages were significantly increased compared to uninfected macrophages. However, there were no significant differences of iNOS expression between rMs064-infected or rMs012-infected macrophages. Lipopolysaccharide (LPS)-treated macrophages were used as the positive control, which showed statistical increases in iNOS expression compared with all the experimental conditions.

### 2.3. Macrophage Surface Molecule Expression

**Figure 4:**
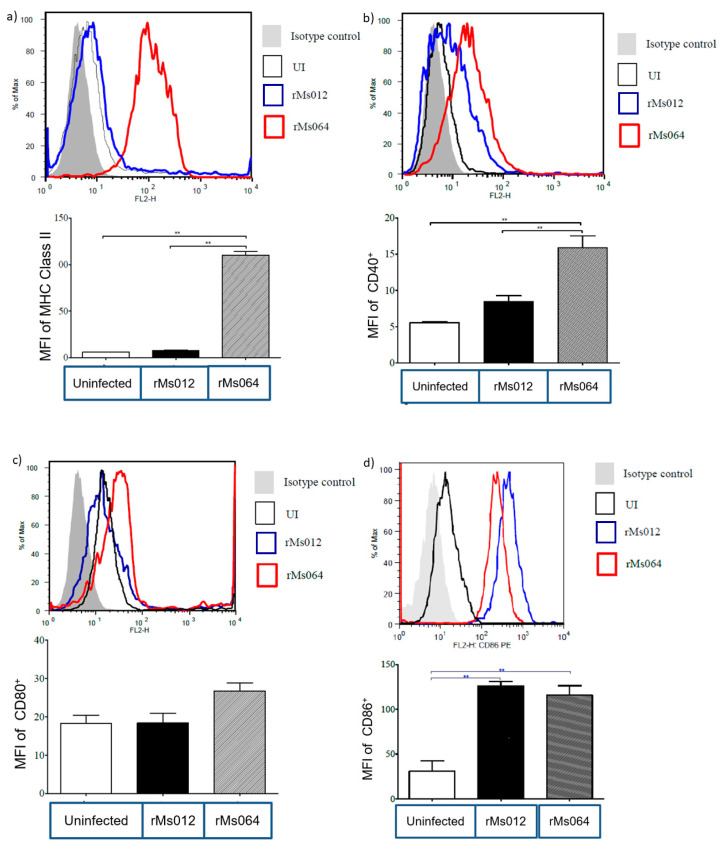
Expression of surface activation markers by rMs064- and rMs012-infected macrophages. (**a**) MHC-II, (**b**) CD40, (**c**) CD80 and (**d**) CD86. Flow cytometry. Values are mean median fluorescence intensity; MFI ± SEM of three independent experiments. Statistical analysis was performed by one-way analysis of variance (ANOVA) followed by the Bonferroni multiple comparison test. ** *p* < 0.001.

Surface expression levels of MHC-II in rMs064-infected macrophages were significantly increased (110 ± 4.00 median fluorescence intensity; MFI), compared to rMs012-infected macrophages (7.68 ± 0.31 MFI) and uninfected macrophages (6.08 ± 0.15 MFI) (Figure 4a). Similarly, CD40 surface expression levels were significantly higher in rMs064-infected macrophages (15.9 ± 1.64 MFI) compared to rMs012-infected (8.44 ± 0.4 MFI) and uninfected macrophages (5.54 ± 0.15 MFI) (Figure 4b). On the other hand, the CD80 expression level did not show statistical differences (Figure 4c). In contrast, Figure 4d shows a significant expression of CD86 in rMs064-infected (115.7 ± 10.68 MFI) and rMs012-infected (126.0 ± 5.0 MFI) macrophages, compared to uninfected macrophages (30.70 ± 11.69 MFI).

### 2.4. Cytokine Production

**Figure 5:**
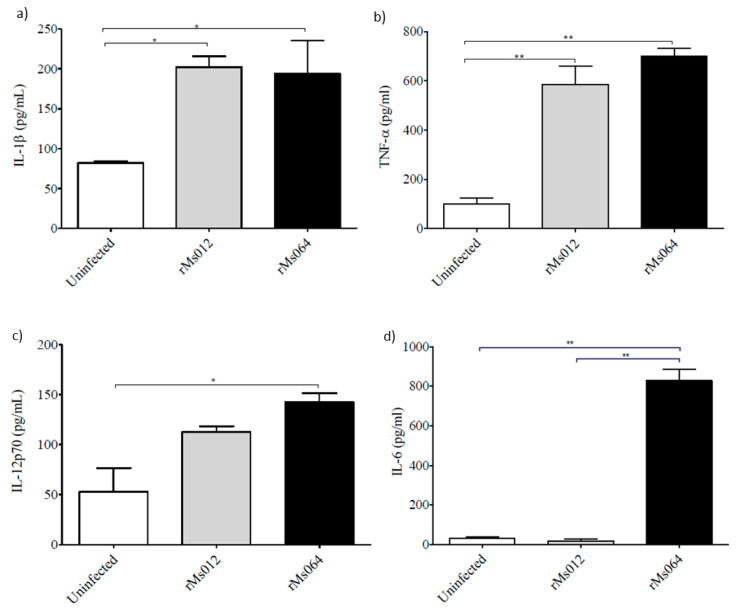
Cytokine production by rMs064 or rMs012-infected macrophages. (**a**) IL-1β; (**b**) TNF-α; (**c**) IL-12; and (**d**) IL-6. Data are presented as the mean concentration of cytokine ±SEM from three independent experiments. Statistical analysis was performed by one-way analysis of variance (ANOVA) followed by the Bonferroni multiple comparison test. * *p* < 0.05 and ** *p* < 0.001.

The production of pro-inflammatory cytokines IL-1β and TNF-α were significantly elevated in both rMs012 and rMs064 infected macrophages compared to uninfected macrophages (Figure 5a,b). rMs064-infected macrophages showed significant increases in the production of IL-12p70 compared to uninfected cells (Figure 5c). IL-6 was significantly increased in rMs064-infected macrophages compared to uninfected and rMs012-infected cells (Figure 5d).

## 3. Discussion

*M. smegmatis* is a fast growing saprophyte found in soil [23]. This nonpathogenic mycobacteria species is commonly used as a surrogate *M. tuberculosis* model to study the physiology, antigenic diversity, and immune responses to pathogenic mycobacteria [23,24,25]. *M. smegmatis* is unable to escape intracellular killing, and its survival is relatively short in the host cell microenvironment [26]. Therefore, *M. smegmatis* is efficiently processed by infected cells, altering the cell surface phenotype and trigger the production of important pro-inflammatory cytokines [27]. Wild type and recombinant *M. smegmatis* possess non-pathogenic effects in natural killer cell or T cell deficient mice [28]. In addition, immunogenic materials produced from mycobacteria are active adjuvants that demonstrate their immunostimulatory activity on the antigen presenting cells (APCs) through interaction with PRR such as TLR-2 and TLR-4 [29]. Thus, *M. smegmatis* may have potential to be used as a live vector for development of TB candidate vaccines.

Macrophages are one of the most important innate immune cells in combating *M. tuberculosis* infection [29]. Mycobacteria and host macrophages interaction is a complex process which can result in several potential outcomes such as bacterial clearance, unchecked multiplication (producing active TB) or latent infection [29]. In vitro macrophages culture is an appropriate approach to study interactions involving mycobacteria and the host cells. This approach make it feasible to elucidate the signaling mechanisms and downstream innate immune activation in early stages of mycobacterial infection [30,31].

The stimulation of innate immunity is of paramount importance in vaccine development. Multiple strategies such as the use of adjuvants and live vectors, including *M. smegmatis* expressing heterologous antigens of vaccine interest, are used to stimulate efficient innate immune responses, resulting in the stimulation of the specific immune response [32,33,34,35,36].

The current study attempted to determine the immune activating effect on J774A.1 murine macrophages of rMs064, which previously demonstrated the induction of specific humoral and cellular immune responses in mice [21].

Upon co-incubation of rMs064 with J774A.1 macrophages in the absence of exogenous stimuli, initial experiments were set up to evaluate the phagocytic behavior and bacilli load. After infection, a reproducible infection rate was observed. This result is in line with an earlier study of *M. smegmatis* infection in murine macrophages [37]. Murine macrophages J774A.1 have been reported to internalize non-pathogenic mycobacteria efficiently in a short time compared with human derived macrophage cell line, THP-1 [38]. In the present study, there were no significant differences on the phagocytic index between rMs064 and rMs012 infected macrophages. The capacity of the vaccine candidate to be internalized by the macrophages is an advantage as it is the first step in the macrophage activation [39].

Despite that the expression of iNOS and the production of NO is not a surrogate of vaccine efficacy, it was evaluated in our study as an evidence of macrophage activation induced by the infection with the vaccine candidate.

The current study showed that rMs064 and rMs012 increased macrophage activity and produced higher iNOS amounts compared to untreated cells. This is similar to a report which demonstrated that *M. smegmatis* induced the expression of iNOS as early as 15 min after infection [37].

The results of phagocytic activity, and iNOS production from rMs064 infected macrophages showed no significant increase compared to rMs012. In the experimental conditions tested, it was not possible to demonstrate a role of Ag85B epitope expression on the macrophage based on these parameters. These results could be explained by the strong intrinsic stimulatory capacity of *M. smegmatis*, that possibly mask the effect of Ag85B expression [40,41,42].

The efficient activation of the specific immune response by the APCs relies on two important processes: the expression of MHC class I and II and co-stimulatory molecules and the production of pro-inflammatory cytokines, which provide the optimal required signals for T-cell activation [43]. To evaluate the immunostimulatory capacity of rMs064, we evaluate the expression of MHC class II and co-stimulatory molecules, together with the determination of a group of cytokines involved in the elicitation of Th1 responses, which are advocated to have a relevant role in the protection against TB [39].

After internalization of antigens through phagocytosis, activated macrophages display peptide–MHC complexes, co-stimulatory molecules and produce inflammatory cytokines as initial signals to T-cell activation [44,45]. Infecting microbes are capable to induce the activation of APCs, which display MHC-II and costimulatory molecules, such as, CD40, CD80 (B7-1) and CD86 (B7-2) among others [46,47,48,49]. The level of costimulatory molecules expression can affect the course of infection and the persistence of intracellular bacteria. The bacteria’s capacity to simulate the expression of these molecules consequently dictates the modulation of immune responses and their survival in the infected host [50,51].

The results of the present study demonstrated the expression of MHC class II and co-stimulatory molecules and enhanced production of pro-inflammatory cytokines after macrophages were infected with rMs064, which is an advantage for its use as a vaccine candidate. It was evident the enhanced expression of MHC-II and CD40 molecules in rMs064 infected macrophages 24 h post-infection, compared to non-infected and rMs012 infected macrophages. No significant differences of CD80 (B7.1) and CD86 (B7.2) expression between rMs064 and rMs012 infected cells were demonstrated, although the level of expression in the case of CD86 was significantly increased compared to uninfected cells. The significant increase in MHC-II in rMs064 infected macrophages could favor antigen presentation. The processing of exogenous antigens and its presentation, results in the binding of peptide–MHC II complexes by the T-cell receptor (TCR) on the surface of APCs, which is a first signal for T cell activation [52]. The increased expression of CD80 (B7.1) and CD86 (B7.2), induced by rMs064, represent the second signal for T cell activation, which together with the increased expression of CD40 lead to further stimulation of the infected APCs upon interaction with the CD40L of the engaged T cell [53]. The increased expression of CD40, CD54, CD80, CD86, and MHCII in DC2.4 dendritic cell line has been reported after infection with *M. smegmatis* and *M. bovis* BCG expressing an epitope from ovalbumin [27].

A significant increase in the production of IL-12p70 was induced in rMs064-infected macrophages. Similar results were obtained on BMDM, BMDCs and THP-1 human monocyte cells infected with *M. smegmatis* [54]. IL-12 is necessary and important for optimal differentiation and regulation of IFN-γ, which is required for Th1 cell activation[55]. Intranasal inoculation of a recombinant *M. smegmatis* viable vaccine expressing sIL-12/granulysin induced elevated levels of serum IFN-γ, IL-12 and IgG2a [56].

A higher level of IL-6 was produced in rMs064-infected macrophages compared to rMs012- or uninfected- macrophages. The difference in the production of IL-6 induced by rMs064- and rMs012- infected J774A.1 macrophages, could be due to the role of the Ag85B epitopes expressed by rMs064. A previous study showed the regulatory effect of Ag85B expression in the production of different cytokines [57]. IL-6 is an important promoter of Th1 immune responses [58,59,60,61,62,63], which indicate the potential of rMs064 to induce this kind of immune response.

rMs064 and rMs012 infected macrophages showed enhanced IL-1β and TNF-α production which are cytokines involved in the induction of Th1 immune responses [62]. In concordance with the results of the current study, murine macrophages infected with *M. smegmatis* and *M. fortuitum* have been found to induce strong pro-inflammatory cytokines such as TNF-α and IL-12 [64].

The abundance of pathogen associated molecular patterns (PAMPs), with rich lipid composition on *M. smegmatis* are potential targets for pathogen recognition receptors (PRRs) [65]. The recognition of PAMPs by host cells activates multiple signaling pathways, which in turn induce the expression of costimulatory molecules and enhanced production of inflammatory cytokines.

rMs064 was capable of inducing the activation of J774A.1 murine macrophages, showing a significant expression of MHCII, CD86 and CD40 molecules and production of IL-1β, TNF-α, IL-12p70, and IL-6. The expression of Ag85B epitopes by rMs064 results in the activation of J774A.1 murine macrophages, with a Th1 inducing profile, which is in agreement with our previous results after the immunization of mice with this experimental vaccine candidate, which induced specific humoral and cellular immune responses with a Th1 pattern [66,67,68]. The results obtained with rMs064 in this and previous in vivo studies supports its further evaluation as a potential TB vaccine.

## 4. Materials and Methods

### 4.1. Macrophage Cell Line

Mouse macrophages cell line J774A.1 (ATCC#TIB-67) was purchased the from American Type Culture Collection (ATCC, Manassas, VA, USA). The cell line was maintained in complete Dulbecco’s Modified Eagles Medium (DMEM) (Invitrogen, Carlsbad, CA, USA) supplemented with 2 mM L-glutamine, 1mM sodium pyruvate 10% fetal bovine serum, penicillin and streptomycin (Thermo Fisher Scientific, Waltham, MA, USA). The cells were grown at 37 °C in a humidified incubator with 5% CO_2_. Upon infection, J774A.1 macrophages were seeded in a 6-well plate containing 2 × 10^5^ cells/mL in antibiotic free media and incubated overnight at 37 °C.

### 4.2. Mycobacterium Smegmatis Strains and Infection Procedure

Non-transformed M. smegmatis mc^2^155 strain [69], and recombinant variants, expressing Ag85B epitopes (rMs064) and transformed with the empty plasmid vector (rMs012) [21] were used in the study. The recombinant strains were grown in Middlebrook 7H9 broth (BD Biosciences, Franklin Lakes, NJ, USA) supplemented with 0.5% glycerol and Tween 80 (Merck, Kenilworth, NJ, USA), respectively, 10% Middlebrook oleic acid dextrose catalase (OADC) enrichment medium (BD Biosciences, Franklin Lakes, NJ, USA) and 10 µg/mL kanamycin. rMs064 and rMs012 cultures in exponential growth phase were centrifuged at 5000× *g* for 10 min and washed twice in phosphate buffered saline (PBS) pH 7.4. Bacterial pellets were re-suspended in DMEM medium. Clumps of bacteria were removed by ultrasonic treatment of bacterial suspensions in an ultrasonic water bath for 5 min. The suspension was then passage through a 23-gauge syringe needle to disrupt remaining bacterial clumps and adjusted to a final OD600: 0.1 after confirming the presence of single cell suspensions by light microscopy. Macrophages were infected with rMs012 or rMs064 (multiplicity of infection; MOI of 10:1) or left untreated. After 4 h of infection, each well was washed 3 times with sterile PBS to remove non-internalized bacteria. Then, the media with penicillin and streptomycin were added to the infected macrophages and left for 24 h at 37 °C.

### 4.3. Phagocytic Assay

The phagocytic activities of macrophages were determined by the number of ingested rMs by each cell. The pulse phase of the experiment (period of co-incubation of macrophage and mycobacteria) was set up at 4 h [70]. Briefly, non-ingested bacilli were removed, and infected cells were washed twice with sterile PBS at 150× *g* for 5 min. The pellet was suspended in 1 mL of PBS, and 10 µL of cell suspension was smeared and fixed on a slide for Ziehl–Neelsen acid fast staining (Thermo Fisher Scientific, Waltham, MA, USA). Slides were observed by microscopy, and the number of phagocytosed bacilli counted. Phagocytic index (PI) was determined as the relative population of the rMs in 100 macrophages [71].

### 4.4. Determination of NO_2_ Concentration and iNOS Assay

The determination NO_2_ in cells supernatants was carried out using the quantitative Nitric Oxide Assay Kit (Thermo Fisher Scientific, Waltham, MA, USA), following the instructions by the manufacturer_._ The determination of iNOS was made as follows: Cells were lysed on ice in lysis buffer (1% NP-40, 50 mN Tris-HCl, pH 7.6, 150 mM NaCl and 2 mM EDTA) and clarified by centrifugation at 5000× *g* for 5 min in a microcentrifuge at 4 °C. In addition, LPS infected macrophages were used as the positive control for this experiment. Proteins were separated by SDS-PAGE and transferred to polyvinylidine fluoride (PVDF) membranes (Biorad, Hercules, CA, USA). Antibodies used were rabbit anti-iNOS (Cell Signaling, Danvers, MA, USA) and goat anti-actin (Abcam, Cambridge, UK). Chemiluminescence was detected using the enhanced chemiluminescent (ECL) reagents (GE Healthcare, Chicago, IL, USA). To determine iNOS expression, densitometry was performed using Adobe Photoshop.

### 4.5. Immunofluorescent Staining of Cells

After the infection procedure, macrophages were stained using fluorescence conjugated antibodies CD40 (Cat. number 124609), CD80 (B7.1) (Cat number 104707), CD86 (B7.2) (Cat. number 159203) (all from Biolegend, San Diego, CA, USA) and MHC Class II (Cat. Number 12-5321, eBioscience, San Diego, CA, USA) and their isotype-matched controls. Live/Dead Fixable Far Red Dead Cell stain (Thermo Fisher, Waltham, MA, USA) were used for distinguishing live from dead cells. Live/dead viability staining and surface staining were done for 30 min at 4 °C. Cells were washed and fixed in 200 µL staining buffer containing 1% paraformaldehyde. Flow cytometry data were obtained using fluorescense-activated cell sorting; (FACS) Canto, (BD Biosciences, Franklin Lakes, NJ, USA) and analyzed by FlowJo software (Tree Star, Woodburn, OR, USA). Flow cytometry data are presented as percentage cell population positive for respective fluorochrome-labelled anti-mouse antibodies normalized with suitable isotype-matched controls. Single cell population was gated by forward scatter versus height and side scatter for size and granularity, and dead cells were excluded.

### 4.6. Cytokines Assay

The production of IL-1β, TNF-α, Il-12p70 and IL-6 were measured in culture supernatants of uninfected and rMS infected macrophages using ELISA Ready-SET-Go!^®^ IL-1β, TNF-α, Il-12p70 and IL-6 kit (eBioscience, San Diego, CA, USA). Briefly, Corning™ Clear Polystyrene 96-Well Microplates (Thermo Fisher, Waltham, MA, USA) were coated with 100 µL of capture antibody (anti mouse IL-1β, TNF-α, IL-12p70 and IL-6) in coating buffer. The plates were sealed and incubated overnight at 4 °C. Then, the plates were washed 5 times with 250 µL washing buffer. The plates were blotted on absorbent paper to remove any residual buffer before being blocked with 200 µL of assay diluent and incubated at room temperature. After 1 h incubation, the plates were washed 5 times for 10 min. Following washes, 50 μL of culture supernatants was incubated 2 h at room temperature. Plates were washed 5 times with 300 μL of wash buffer and 100 μL of substrate solution was added. Plates were incubated for 30 min at room temperature. Finally, 25 µL of stop solution was added into each well and the absorbance was read at 450 nm in a microplate reader (Tecan, Mannedorf, Switzerland). Uninfected cells were used as a control. Cytokine concentrations (pg/mL) were determined using a standard curve based on the standard positive control provided by the manufacturer.

## 4.7. Statistical Analysis

Statistical analysis was performed by one-way analysis of variance (ANOVA) followed by the Bonferroni multiple comparison test (Prism, GraphPad Software Inc., San Diego, CA, USA) for determination of significant differences between groups (* *p* ≤ 0.05) and (** *p* ≤ 0.001).
